# P-392. Low-Barrier Open Access HIV Care Clinic in New Orleans, Louisiana

**DOI:** 10.1093/ofid/ofaf695.609

**Published:** 2026-01-11

**Authors:** Tat Yau, Eric Babineaux, Lauren Richey

**Affiliations:** LSU Health New Orleans School of Medicine, New Orleans, LA; LSU Health New Orleans, New Orleans, Louisiana; LSU Health Sciences Center New Orleans, New Orleans, Louisiana

## Abstract

**Background:**

There are many barriers to ending the HIV epidemic. Substance use, mental illness, housing instability, and providers shortages contribute to the challenges of achieving the 95-95-95 targets. We present our novel experience in implementing a low-barrier, open-access clinic to address barriers to HIV care in New Orleans, Louisiana using End the HIV Epidemic funding.

Table 1 Demographics and Psychosocial Risk Factors of Patients Attending the Open Access Clinic

**Figure:**
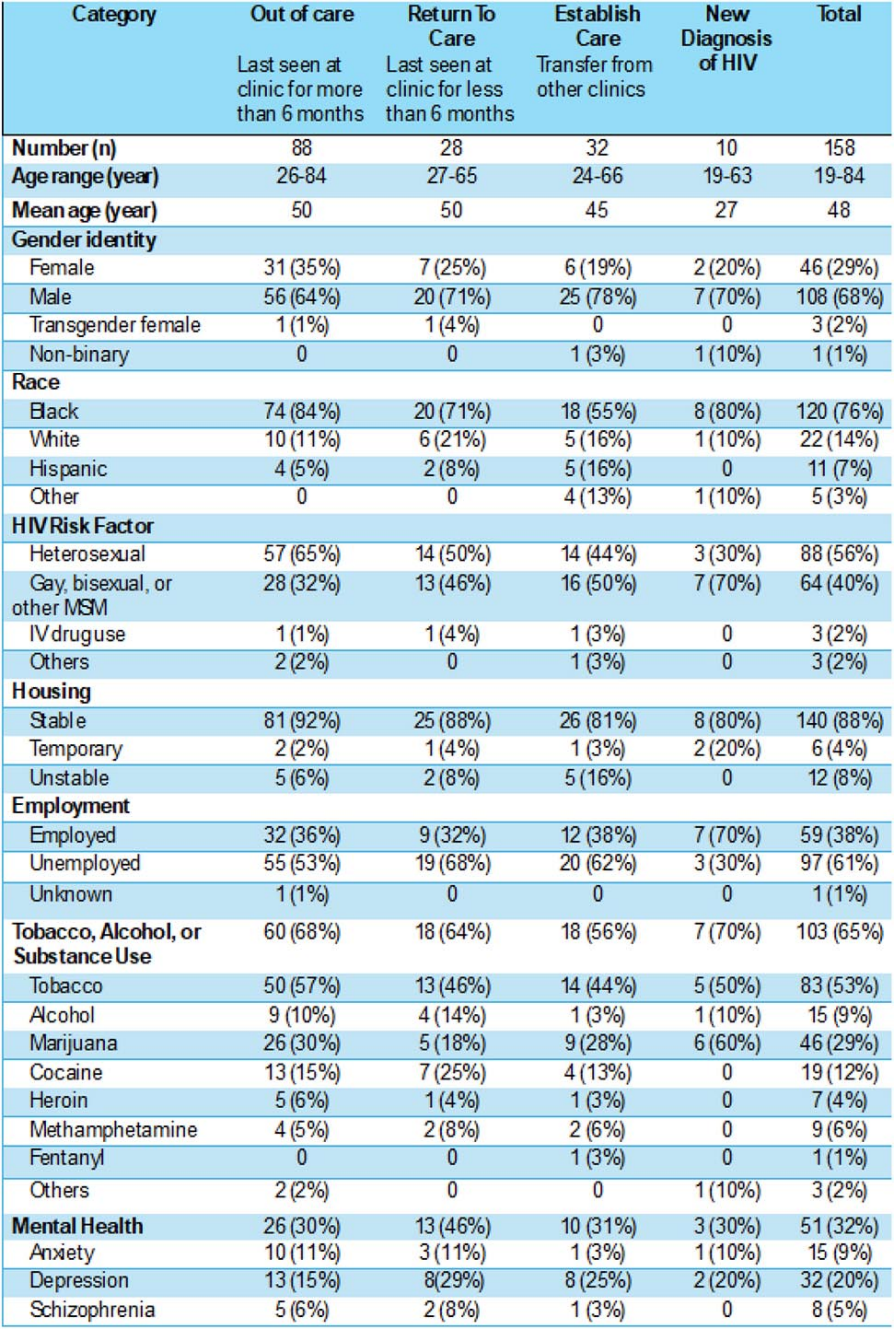

**Methods:**

A twice-weekly walk-in clinic with an open schedule, complemented by a twice-weekly evening clinic, was implemented at the HIV Outpatient Program (HOP) at University Medical Center New Orleans (UMCNO) to accommodate patients who require appointments with flexibility. This initiative enables close follow-up for patients recently discharged from the hospital, immediate appointments for out-of-care patients seeking walk-in services, immediate consultations for newly diagnosed patients, and urgent visits for current primary care patients. Our goals is to enhance linkage and entry and re-entry to care, optimize engagement and retention in care, and, consequently, improve viral suppression for patients who are difficult to engage.

**Results:**

158 patients visited our low-barrier clinic from August 2024 to April 2025. Patients were classified into four categories: new diagnosis of HIV, establishing care (from another clinic), returning to care (within 6 months), or out of care (more than 6 months). Most patients were unemployed (61%) and had a current history of tobacco, alcohol, or substance use (65%). Additionally, 12% had temporary or unstable housing and 32% had a mental health diagnosis. There were 63 (40%) with a detectable viral load. Most of the patients who had follow-up labs after engaging with the clinic showed viral suppression (82%) or significant decrease in viral load (11%).

**Conclusion:**

The implementation of a low-barrier open-access clinic demonstrated promising improvement in viral load by enhancing access for traditionally hard-to-reach patients.

**Disclosures:**

Eric Babineaux, DNP, AAHIVS, Gilead: Contract speaker

